# Contribution of *Drosophila* TRPA1 to Metabolism

**DOI:** 10.1371/journal.pone.0152935

**Published:** 2016-04-07

**Authors:** Jung-Eun Lee, Yunjung Kim, Kyoung Heon Kim, Do Yup Lee, Youngseok Lee

**Affiliations:** 1 Department of Bio and Fermentation Convergence Technology, BK21 PLUS project, Kookmin University, Seoul 02707, Korea; 2 Department of Biotechnology, Graduate School, Korea University, Seoul, Korea; National Research Council of Italy, ITALY

## Abstract

Transient receptor potential (TRP) cation channels are highly conserved in humans and insects. Some of these channels are expressed in internal organs and their functions remain incompletely understood. By direct knock-in of the *GAL4* gene into the *trpA1* locus in *Drosophila*, we identified the expression of this gene in the subesophageal ganglion (SOGs) region. In addition, the neurites present in the dorsal posterior region as well as the drosophila insulin-like peptide 2 (dILP2)-positive neurons send signals to the SOGs. The signal is sent to the crop, which is an enlarged organ of the esophagus and functions as a storage place for food in the digestive system. To systematically investigate the role of TRPA1 in metabolism, we applied non-targeted metabolite profiling analysis together with gas-chromatography/time-of-flight mass spectrometry, with an aim to identify a wide range of primary metabolites. We effectively captured distinctive metabolomic phenotypes and identified specific metabolic dysregulation triggered by TRPA1 mutation based on reconstructed metabolic network analysis. Primarily, the network analysis pinpointed the simultaneous down-regulation of intermediates in the methionine salvation pathway, in contrast to the synchronized up-regulation of a range of free fatty acids. The gene dosage-dependent dynamics of metabolite levels among wild-type, hetero- and homozygous mutants, and their coordinated metabolic modulation under multiple gene settings across five different genotypes confirmed the direct linkages of TRPA1 to metabolism.

## Introduction

The gastrointestinal tract is a digestive organ system responsible for controlling ingestion, digestion, absorption, and excretion. The regulation of these processes requires a proper interplay between the intrinsic and extrinsic neuronal pathways. The intrinsic pathway is controlled by the enteric nervous system embedded in the wall of the gut. Furthermore, the extrinsic pathway is mediated by afferent nerve fibers that transfer sensory information to the central nervous system (CNS). The sensory integration in the CNS via the visceral afferent fibers is essential for the coordination of the gut reflex behavior, which is intrinsically controlled [1].

Organisms, from flies to human beings, have a similar digestive system that maintains homeostasis [2]. Extracellular digestion takes place in an internal pouch or tube; however, intracellular digestion occurs within the cells. Digestive tubes typically process nutrients in the following five steps: mechanical processing, secretion of enzymes, enzymatic hydrolysis, absorption, and elimination. Herbivorous insects such as the fly chew plants and break food into small particles with the help of the proboscis. From the mouth, the food passes through the pharynx, where salivary secretions start to mix the small particles, before they go into the esophagus and pass into the crop.

About 20 of the 30 mammalian transient receptor potential (TRP) channels are expressed within the digestive system. They are believed to have important functions with regard to taste, chemesthesis, pain, hyperalgesia, and mechanosensation and are involved in aiding gastrointestinal motility, absorptive and secretory processes, and maintenance of mucosal homeostasis [1]. The drosophila TRP superfamily is composed of 11 TRP channels that can be subdivided into 5 subfamilies: 3 TRPCs (TRP, TRPL, TRPγ), 2 TRPVs (IAV, NAN), 1 TRPM (dTRPM), 1 TRPN, and 4 TRPAs (TRPA1, PAIN, PYX, WTRW) [3]. In addition, genes for the 2 distantly related subfamilies TRPP (AMO) and TRPML exist in the fly genome. However, there is no known TRP channel in the digestive system of *Drosophila* for which a role has been elucidated, while most TRP channels, except TRPP and TRPML, have roles in neurogastroenterology, as determined in mammalian studies [1].

By inducing a *GAL4* reporter knock-in to the *trpA1* locus, we found that TRPA1 is highly expressed in the dorsal posterior region [4]. These neuronal clusters send neurites to the SOG area, which is known to control digestion. In addition, neurites from the SOG relay signals to the crop, which plays an important role in storing foods. This neuronal pathway may have a vital function in controlling the digestive system. However, no study has examined the metabolomic changes in the TRP mutants in mammals as well as in *Drosophila*. Here, we provide the first evidence of the metabolic changes in TRPA1 mutants on a systemic level.

To address the role of TRPA1 in metabolism, we applied non-targeted profiling analysis of the primary metabolites together with gas chromatography/time-of-flight mass spectrometry (GC-TOF MS) [5]. We effectively captured the distinctive metabolomic phenotypes and the specific metabolic dysregulation triggered by the TRPA1 mutation. Integrative statistical analysis revealed the direct effect of TRPA1 mutation on methionine salvage pathway and free fatty acid metabolism and confirmed it by exploring the metabolic dynamics under multiple gene settings, including *trpA1*^*1*^, *UAS*-*trpA1*;*trpA1*^*GAL4*^, and *trpA1*^*GAL4*^, which imply the direct effect of TRPA1 on the metabolism.

## Materials and Methods

### Fly stocks

The *trpA1*^*1*^, *trpA1*^*GAL4*^, and *UAS*-*trpA1* fly lines are described in a previous report [6]. The following fly strains were obtained from the Bloomington Stock Center: 1) *w*^*1118*^, and 2) *UAS*-*mCD8*::*GFP*. The *w*^*1118*^ strain was used as the “wild-type” control. The *trpA1*^*1*^, *trpA1*^*GAL4*^, and *UAS*-*trpA1* strains were outcrossed at least 5 generations in the previous study.

### Immunohistochemistry

Antibody stains of adult brains were performed as previously described [7]. Briefly, dissected brains were stored into 24-well cell culture plates (Costar Corp.) containing 940 μL of fix buffer (0.1 M Pipes pH 6.9, 1 mM EGTA, 1% TritonX-100, 2 mM MgSO_4_, 150 mM NaCl) and 60 μL of 37% formaldehyde. The plates were placed on ice for 30–45 min, depending on the dissecting period. Subsequently, the brains were washed 3 times (1× PBS, 0.2% saponin) and blocked for 8 h at 4°C with 1 mL of blocking buffer (1× PBS, 0.2% saponin, 5 mg/mL BSA). The brains were incubated overnight at 4°C with the primary antibodies (mouse anti-green fluorescence protein (GFP; 1:1,000 Invitrogen-Molecular Probes)), and rabbit anti-dILP2 (1:100), washed 3 times, blocked for 15 min, incubated for 4 h at 4°C with the secondary antibodies (Alexa 488 and Alexa 568; 1:200 Invitrogen-Molecular Probes), and again washed 3 times. The brains were finally stored and mounted into 1.25× PDA solution (37.5% glycerol, 187.5 mM NaCl, 62.5 mM Tris, pH 8.8) and viewed by confocal microscopy (Carl Zeiss LSM510).

### GC-TOF MS analysis for metabolites

Three to seven-day-old male flies (n = 30 for each genotype) were flash-frozen using liquid nitrogen and freeze-dried. The lyophilized flies were ground with a 5-mm i.d. stainless-steel ball using Mixer Mill MM400 (Retsch GmbH & Co., Germany) before solvent extraction (methanol:isopropanol:water, 3:3:2, v/v/v, 750 μl). After centrifugation at 13,200 rpm for 5 min at 4°C, 650 μl of supernatant was collected in fresh 1.5-ml tubes. The aliquots were concentrated to dryness in a speed vacuum concentrator (SCANVAC, Korea), and the resultant dried extracts were kept at -80°C until analysis using GC-TOF mass spectrometry.

Five microliters of 40 mg/ml methoxyamine hydrochloride (Sigma-Aldrich, St. Louis, MO) in pyridine (Thermo, USA) was added to the dried samples and allowed to react for 90 min at 30°C. A mixture of internal retention index (RI) markers was made using fatty acid methyl esters of C8, C9, C10, C12, C14, C16, C18, C20, C22, C24, C26, C28, and C30 in chloroform at concentrations of 0.8 mg/ml (C8–C16) and 0.4 mg/ml (C18–C30). Two microliters of the RI markers and 45 μl of *N*-methyl-*N*-trimethylsilyltrifluoroacetamide (MSTFA + 1% TMCS Thermo, USA) was added and incubated for 1 h at 37°C. The derivatized extracts were injected using an Agilent 7693 ALS (Agilent Technologies, Wilmington, DE) in splitless mode; the split vent was opened after 2 sec. Volatilized metabolites were separated by an Agilent 7890B gas chromatograph (Agilent Technologies, Wilmington, DE) equipped with an RTX-5Sil MS column (Restek, Bellefonte, PA). The oven temperature was programmed at 50°C for 1 min, then ramped at 20°C/min to 330°C, and maintained for 5 min to the end of the run. Mass spectrometry analysis was performed by a Leco Pegasus HT time of flight mass spectrometer with a transfer-line temperature of 280°C and an ion source temperature of 250°C (LECO, St. Joseph, MI). Mass spectra of metabolites were acquisitioned from m/z 85 to 500 at 17 spectra/sec and 1700 eV detector voltage.

### Statistical data analysis

The data sets were normalized by total ion chromatogram signals of all structurally identified compounds. General statistical analysis was performed using *Statistica* software vs. 7.0 (StatSoft, Tulsa, OK) with all continuous variables. Univariate statistics was based on Student’s t-test, and multivariate statistics was performed using unsupervised principal component analysis (PCA). Hierarchical clustering analysis (HCA) and pavlidis template matching (PTM) were conducted by Spearman rank correlation and average linkage methods were built in *Multi Experimental Viewer* (MeV, TIGR).

### Metabolic network construction

The metabolic network was created according to the method developed by Fiehn et al [8]. Briefly, Molefile-encoded chemical structures of all identified metabolites were obtained from PubChem Compound database using compound identifier (CID). The structures were introduced to pairwise Tanimoto chemical coefficient calculations between two compounds in the PubChem structure clustering module. The resultant similarity matrix and the corresponding chemicals were incorporated as an input into Cytoscape network files in.SIF file format. A threshold of 0.7 Tanimoto score was applied for the similarity cut-off value. In addition, the biochemical association between two metabolites, which are connected via one enzymatic reaction was implemented, and the two-layered correlation matrix was imported into Cytoscape. The graph was visualized in an organic layout with some modifications for clear imaging. Fold change was presented as node size, and direction (up/down) was imaged as node color (red/blue resp.) only for metabolites passing the statistical criteria (Student’s t-test, *P*< 0.05).

## Results and Discussion

### Expression pattern of *trpA1* in the *Drosophila* brain

**Fig 1:**
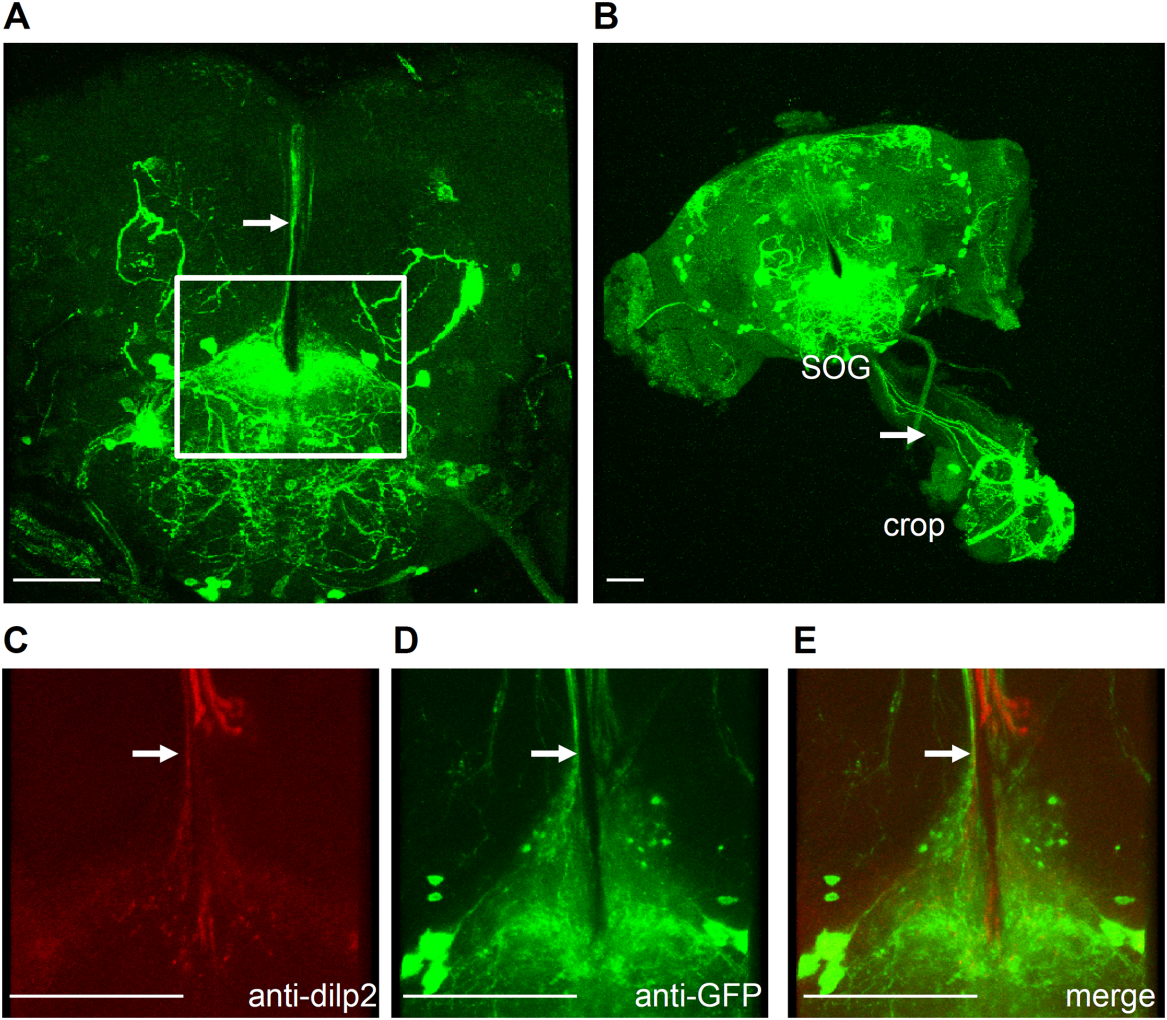
A *GAL4* knock-in to the *trpA1* locus drives the expression in the digestive system. (A-B) Brains dissected and stained with anti-GFP from *UAS*-*mCD8*::*GFP*;*trpA1*^*GAL4*^ flies at the adult stage. (C–E) Brains dissected and stained with anti-dILP2 (C) and anti-GFP (D) from *UAS*-*mCD8*::*GFP*;*trpA1*^*GAL4*^ flies in the adult stage. The merged image is shown (E). Broad expression of the *trpA1* reporter is apparent in the subesophageal ganglions (SOG). Scale bars, 50 m.

*Drosophila* TRPA1 functions in association with chemosensory and thermosensory neurons to avoid the fly’s exposure to unfavorable environmental changes such as increase in temperature (>30°C) and exposure to chemicals such as aristolochic acid, wasabi, and citronellal during its adult stage [9–12]. We recently showed that TRPA1 also has a role associated with circadian pacemaker neurons in adult brain [6]. To identify unknown functions of TRPA1 in adult brain, we first examined the expression of GAL4 reporter in *trpA1*^*GAL4*,^ which introduces the *GAL4* gene at the translation initiation codon site for TRPA1 [9]. Next, we introduced a membrane-tethered GFP (*UAS*-*mCD8*::*GFP*) as a visible marker. Strong synaptic arborizations in SOG area were detected from the combined flies with *trpA1*^*GAL4*^ and *UAS*-*mCD8*::*GFP* (Fig 1A). In addition, the signals from the SOG relay information to the crop in which the foods are stored until necessary (Fig 1B). Furthermore, we previously reported on the newly identified neuronal clusters in the dorsal posterior region [4]. Although we still do not know the function of this cluster, we understand that the neurites send signals to the SOG, where they may have contact with the axons of the dILP2-positive neurons in the pars intercerebralis (Fig 1C–1E note arrow). These results indicate that TRPA1 might have a role in controlling the regulation of the gastrointestinal tract. Thus, it remains to be seen whether TRPA1 mutants would exhibit defects in metabolism.

### Suppression of *trpA1* induces global alteration of metabolic phenotype

GC/MS-based metabolite profiling was performed in 30 samples, which were divided into 5 different genotypes, including 6 replicates for wild-type, *trpA1*^*1*^, *trpA1*^*1*^/+, *UAS*-*trpA1*;*trpA1*^*GAL4*^, and *trpA1*^*GAL4*^, respectively. In total, 109 out of 1200 unique metabolic signatures were structurally identified and quantified by GC-MS. Metabolite identifications were made based on spectral similarities and retention time index using the *BinBase* algorithm, and matched against the *Fiehn* mass spectral library and the NIST05 commercial library (http://fiehnlab.ucdavis.edu/Metabolite-Library-2007/) [13]. The metabolites identified were reported if they were present in at least 80% of the samples per study group [14]. The resultant metabolites covered a range of metabolic pathways, including glycolysis, TCA cycle, pentose phosphate pathway, and nitrogen metabolism.

**Fig 2:**
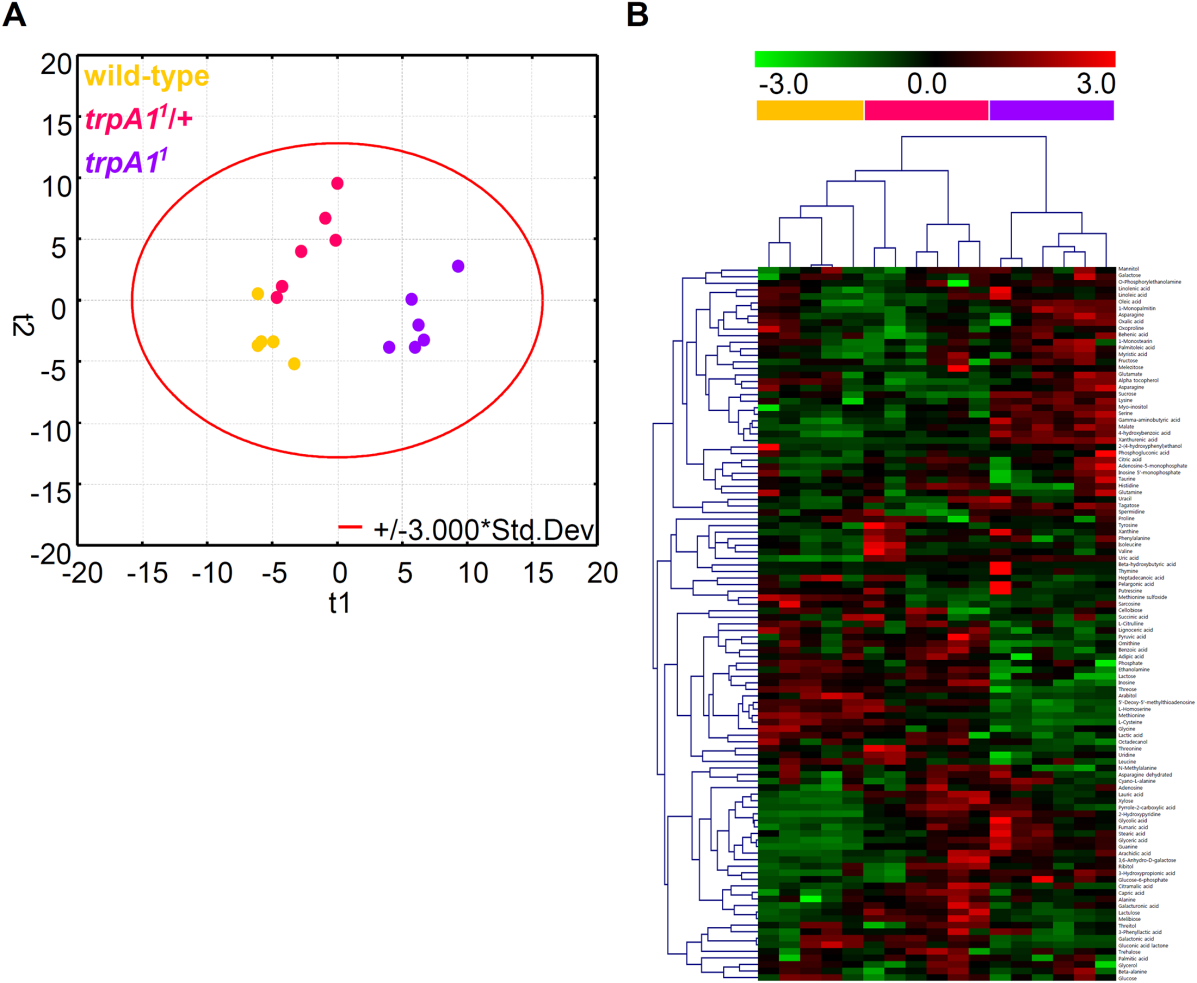
Metabolomic phenotypes integrated with primary metabolic features. (A) Principal component analysis of metabolomic profiles of 109 metabolites of the wild type (orange) and the homozygous mutant (purple). T1 indicates discriminating vector 1 that explained the largest degree of variation in the dataset. Likewise, T2 indicates principal component 2 with the second largest degree of variation. (B) Hierarchical clustering analysis. Clustering analysis was performed across the metabolites and samples by using Spearman rank correlation and average linkage methods. Each column and each row represent a fly sample and an individual metabolite, respectively.

For the first quantitative evaluation, normalized ion intensities for the 109 unique metabolites were analyzed using unsupervised multivariate statistics (principal component analysis, PCA) to globally compare biochemical traits between the *trpA1*-expressed groups (wild-type and heterozygotes, *trpA1*^*1*^/+) and the *trpA1*-suppressed group (homozygotes, *trpA1*^*1*^). The resultant score scatterplot showed that the principal factors discriminated between the metabolite profiles in accordance with the *trpA1* effect. Vector 1, explaining 25.4% of the total variance, mainly discriminated the metabolite profiles of the wild-type and *trpA1*^*1*^/+ from *trpA1*^*1*^ (Fig 2A). With the combination of vector 2 (explaining 16.8% of the total variance), which distinctively clustered the wild-type and the heterozygous group, 42.2% of the total variance was explained among the three different genotypes. The metabolic uniqueness of each genotype was further confirmed by a hierarchical clustering analysis (HCA) using Spearman distance metric [15] and average linkage [16]. The clustering analysis allowed a more detailed insight; certain groups of chemicals showed biochemical relevance with a co-regulatory expression pattern in accordance with the different genotypes (Fig 2B). Altogether, the results implied that the repression of *trpA1* affected a range of primary metabolism, in which the integrative metabolomic phenotype was clearly distinguished while intra-variability within each group was minimized.

Next, we performed univariate statistical analysis (Student’s t-test, *P*<0.05) to firstly interrogate compositional differences in metabolites between the wild-type and *trpA1*^*1*^. A total of 46 compounds (~40%) significantly changed (*P*<0.05) in *trpA1*^*1*^ compared to the wild-type, where 30 were up-regulated and 16 were down-regulated (Table 1), demonstrating the dramatic reorganization of metabolic pathways upon *trpA1* expression. Among them, the highest fold change was found in xanthurenic acid (26.2-fold up-regulation in the mutant). The dramatic increase of xanthurenic acid is an expected observation, given that the metabolite is a lateral reaction product of the ommochrome pathway in the synthesis of eye-pigment. This is a phenotypic characteristic of the knock-in mutants that occurs because of the insertion of *mini*-*white* as a marker [17]. To some extent, this validates technological sensitivity in detecting patterns of metabolite abundance associated with unknown experimental noise (e.g. genetic or phenotypic background). No other direct metabolic signature was induced from the eye pigment in the current experiment (e.g., tryptophan metabolism).

**Table 1:** List of metabolites showing significant difference in *trpA1*^*1*^ compared to the wild-type. *P* values are computed using Student’s t-test (*P*<0.05), and fold changes are calculated by dividing the average of *trpA1*^*1*^ by the average of the wild-type.

Name	*P*-value	Fold change	Name	*P*-value	Fold change
Xanthurenic acid	3.93E-06	26.18	Glycine	0.005	0.91
Guanine	0.000	7.75	Ornithine	0.019	0.86
Pyrrole-2-carboxylic acid	0.040	3.33	Lactic acid	0.001	0.83
Uric acid	1.19E-07	3.19	Inosine	0.000	0.83
Melezitose	0.010	3.08	Lignoceric acid	0.026	0.74
Malate	1.86E-05	2.94	Ethanolamine	0.006	0.73
2-Hydroxypyridine	4.14E-05	2.67	L-Homoserine	1.96E-05	0.68
3-Hydroxypropionic acid	0.002	2.02	Threose	0.000	0.62
Glyceric acid	0.000	2.01	5’-Deoxy-5’-methylthioadenosine	3. 86E-06	0.60
4-Hydroxybenzoic acid	9.56E-05	1.98	L-Cysteine	5. 37E-06	0.50
Citric acid	0.041	1.87	Galactonic acid	0.022	0.48
Glycolic acid	0.032	1.78	Methionine sulfoxide	0.000	0.46
Glucose-6-phosphate	0.036	1.77	Lactose	0.038	0.46
Sucrose	0.004	1.75	Gluconic acid lactone	0.029	0.44
Fumaric acid	0.005	1.71	Arabitol	0.005	0.18
Tagatose	0.001	1.57	Methionine	2.1E-08	0.15
Xylose	0.002	1.45			
Stearic acid	0.020	1.45			
Gamma-aminobutyric acid	0.001	1.44			
Myo-inositol	0.003	1.42			
Fructose	0.034	1.36			
3,6-Anhydro-D-galactose	0.000	1.36			
Lauric acid	0.000	1.28			
Oleic acid	0.036	1.26			
Arachidic acid	0.006	1.24			
Lysine	0.012	1.22			
Serine	0.001	1.16			
1-Monopalmitin	0.020	1.16			
Palmitoleic acid	0.008	1.10			
O-Phosphorylethanolamine	0.014	1.08			

The second highest fold change was observed in a purine nucleobase, guanine, which shows 7.8-fold up-regulation in the *trpA1*-repressed group *trpA1*^*1*^ compared to the wild-type group. Upon following the nucleobase, diverse types of endogenous metabolites, such as pyrrole-2-carboxylic acid, uric acid, melezitose, malate, and 2-hydroxypyridine, presented the most dramatic up-regulation ranging from 3.3–2.7 fold changes (Table 1). Pyrrole-2-carboxylic acid is a degradation product of sialic acid that has been observed in *Drosophila* embryos [18]. Sialic acid is a crucial player in many biological processes, such as the development and functioning of the nervous system [19]; its abnormal metabolism (sialylation) is linked to longevity and temperature-dependent paralysis [18]. Another rare metabolite was melezitose, a non-reducing trisaccharide, which is produced by many plant sap insects in order to reduce osmotic stress by reducing their own water potential [20]. The modulation of the atypical carbohydrate concurred with the up-regulation of an uncommon natural hexoketose, tagatose. This sugar has been used as a dietary supplement for numerous health benefits such as for the promotion of weight loss [21], improvement in chances of pregnancy, and in the alleviation of symptoms associated with type 2 diabetes [22]. The up-regulation of the carbohydrates was further extended to alteration in common sugars such as fructose, sucrose, and glucose-6-phosphate, which implies aberrant activity in a wide range of carbohydrates during metabolism in *trpA1*^*1*^ flies.

In contrast, the intermediates of carbohydrate metabolism such as lactose, arabitol, gluconic acid lactone, and galactonic acid were observed to be among the most drastic down-regulated of the *trpA1*^*1*^ group. Arabitol is a sugar alcohol that is formed as an end product. High levels of arabitol in biofluids is often accompanied by dysfunction of the pentose phosphate pathway (PPP) [23]. The modulated activity of the pathway is evidenced by down-regulation of the gluconic acid, lactone, which is the entry molecule of pentose phosphate pathway. Amongst the down-regulated metabolites, methionine was most significantly reduced in the *trpA1*^*1*^ group (0.15 fold changes relative to the wild-type). This is an essential sulfur-containing amino acid and a precursor of S-adenosylmethionine (SAM), and is related with increased BMI and cardiovascular disease with high dietary intake [24]. Suppression in other amino acids was detected in amino acids, glycine, cysteine, and homoserine.

### Reconstructed metabolic network revealed that methionine salvage pathway and lipid metabolism were directly linked to *trpA1* expression

**Fig 3:**
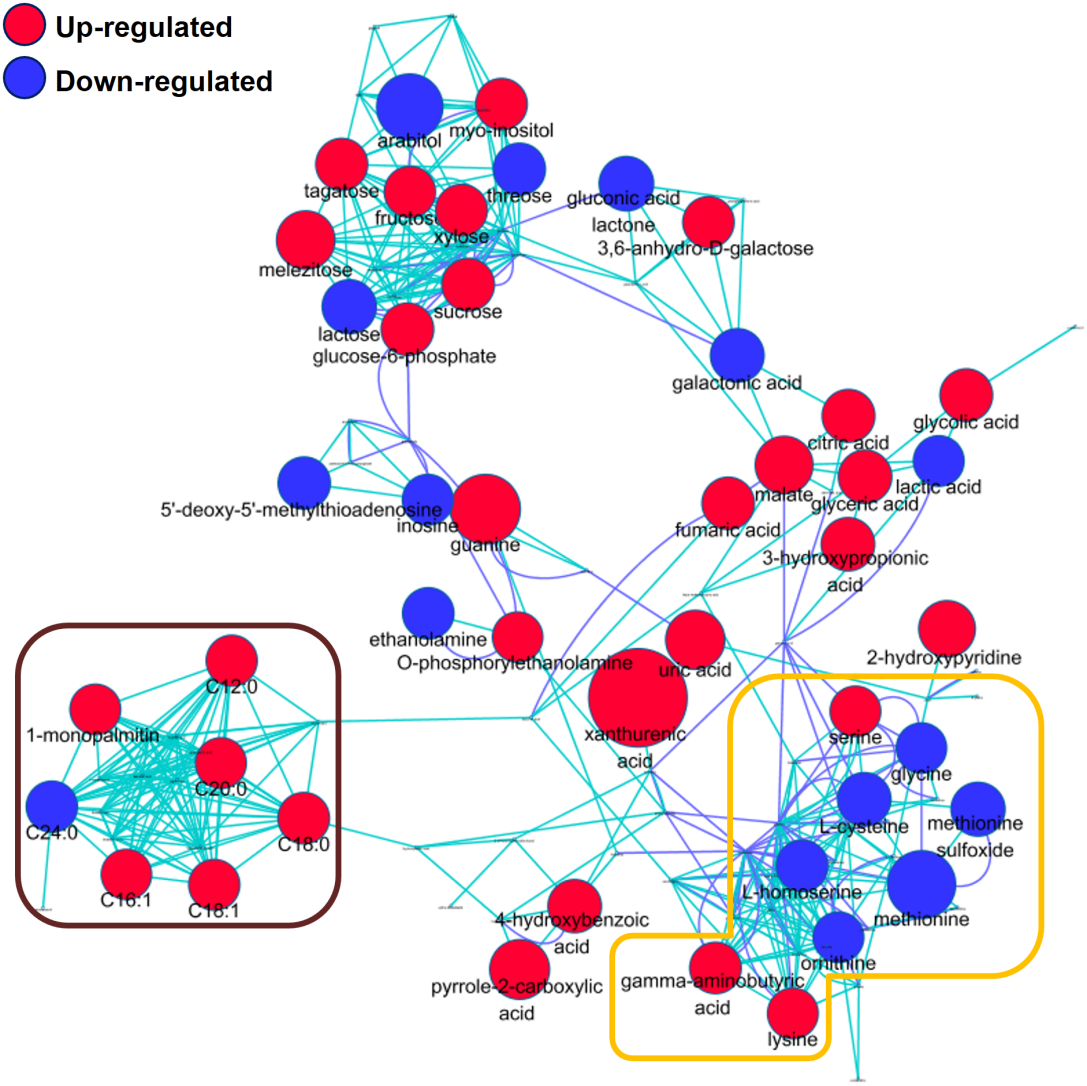
Metabolic networks of biochemical reaction pairs and chemical similarity. The regulation of all identified metabolites in files is depicted. Blue = down-regulated metabolites, red = up-regulated metabolites in the heterozygous mutants (*trpA1*^*1*^) compared to the wild-type (Student’s t-test, *P*<0.05). Node sizes reflect the magnitude of differential metabolite expression. Metabolites that did not show significant difference in levels were left unnamed to maintain visual clarity. Dark blue edges represent connections determined via Kegg reaction pair information, and light blue edges represent assemblies as evaluated using Tanimoto scores (score > 0.7)

For a systematic view of metabolic modulation at the levels of a biochemical network, we explored the coordinated network structure of metabolite dynamics. We used a multi-layered metabolic network that was constructed on the basis of biochemical relevance and chemical structure similarity [8,25,26]. The newly organized metabolic structure comprehensively mapped novel metabolites, including the important carboxylic acid and pyrrole-2-carboxylic acid as well as lipids such as 1-monostearin and 1-monopalmitin. Subsequently, we contrasted the metabolic regulation structures of *trpA1*-deleted mutants in comparison to the wild-type (Fig 3).

The resultant metabolic map provided a characteristic snapshot that coordinated similar expressional alteration with their biochemical (and/or chemical structural) relevance. First, the concomitant down-regulation was captured in amino acid clusters (Fig 3. orange box), including glycine, cysteine, and methionine, as depicted in the univariate statistics where the perturbation propagated to methionine sulfoxide and homoserine within the cluster. Methionine sulfoxide is an oxidation product of methionine that is reversibly converted to its reduced form by methionine sulfoxide reductase, and hence, the endogenous level of the metabolite is regarded as a molecular indicator of oxidative stress in vivo [27]. The metabolic modification was further extended to 5'-methylthioadenosine (MTA), a sulfur-containing nucleoside and key molecule of the methionine salvage pathway, which was concurrently down-regulated among the other nucleosides. MTA is a by-product in the synthesis of polyamines and originates from S-adenosylmethionine that is formed by the condensation of methionine with ATP [28]. In accordance with that, a polyamine, ornithine, was observed in lower expression levels as compared to the wild-type while other polyamines, putrescine and spermidine, showed relatively constant levels.

Of interest, the snapshot of the synchronized metabolic modulation in *trpA1*-deleted mutants mirrored the dietary restriction-induced metabolic physiology, and is known to promote longevity [29]. It has been reported that specific nutrients such as amino acids influence longevity; in particular, dietary restriction involving reduced intake of casein, a major amino acid source, extends the lifespan of *Drosophila* [30]. Specifically, methionine has been proposed as the single key factor modulating lifespan and fecundity where dietary restriction promotes longevity but impairs reproduction [31]. The beneficial effects of methionine restriction were also found in mammals, including rats and mice [32,33]. The methionine restriction-driven lifespan required a low amino-acid status [34] and a specifically low cysteine level [35]. In consistency with that, our results revealed lower levels of amino acids, particularly, sulfur amino acids and sulfur-containing metabolites in *trpA1*^*1*^, as demonstrated in the snapshot of the metabolic network. It has been reported that TRPA1 is activated by extracts from garlic that contain various organosulfur compounds [36], implying the potential association between TRPA1 and sulfur-containing metabolites and the association of the gene with metabolic pathways (e.g., methionine salvage pathway) and physiology (e.g., lifespan and reproduction). This possible correlation between *trpA1* deficiency and extended lifespan needs to be further validated by analyzing fly lifespan, which was performed by another group (personal communication with Dr. Joong-Jean Park in Korea University as well as his poster presentation in the 2^nd^ Asia-Pacific Drosophila Research Conference, *manuscript prepared*).

The strong link between expression pattern and biochemical relevance was also discerned in the lipid cluster (dark brown box). A range of free fatty acids also showed increased contents in lauric acid (C12:0), palmitoleic acid (C16:1), stearic acid (C18:0), oleic acid (C18:1), and arachidic acid (C20:0). The dysregulation was further intensified in neutral lipid metabolism (1-monopalmitin). In contrast to the overall up-regulation in the contents of fatty acids, a very long chain fatty acid, lignoceric acid, showed low abundance. Very long chain fatty acids (VLCFAs) are exclusively oxidized in peroxisomes and their levels are significantly increased in the tissues of patients with peroxisomal disorders [37]. The consequential modulation in the lipid metabolism was proposed in association with a sulfur amino acid, cysteine, which is driven by the methionine-restricted condition [35].

Other clusters were found in the sugar/sugar alcohol intermediates. The clusters showed relatively less direct functional linkage, but the majority of the carbohydrates significantly increased, except lactose and threose. The physiological implications of TRPA1 in sugar/sugar alcohol intermediates need to be explored by further in connection with functional studies.

### Confirmation of *trpA1* effects on the *Drosophila* metabolism by gene-dosage effect and multiple genetic settings

**Fig 4:**
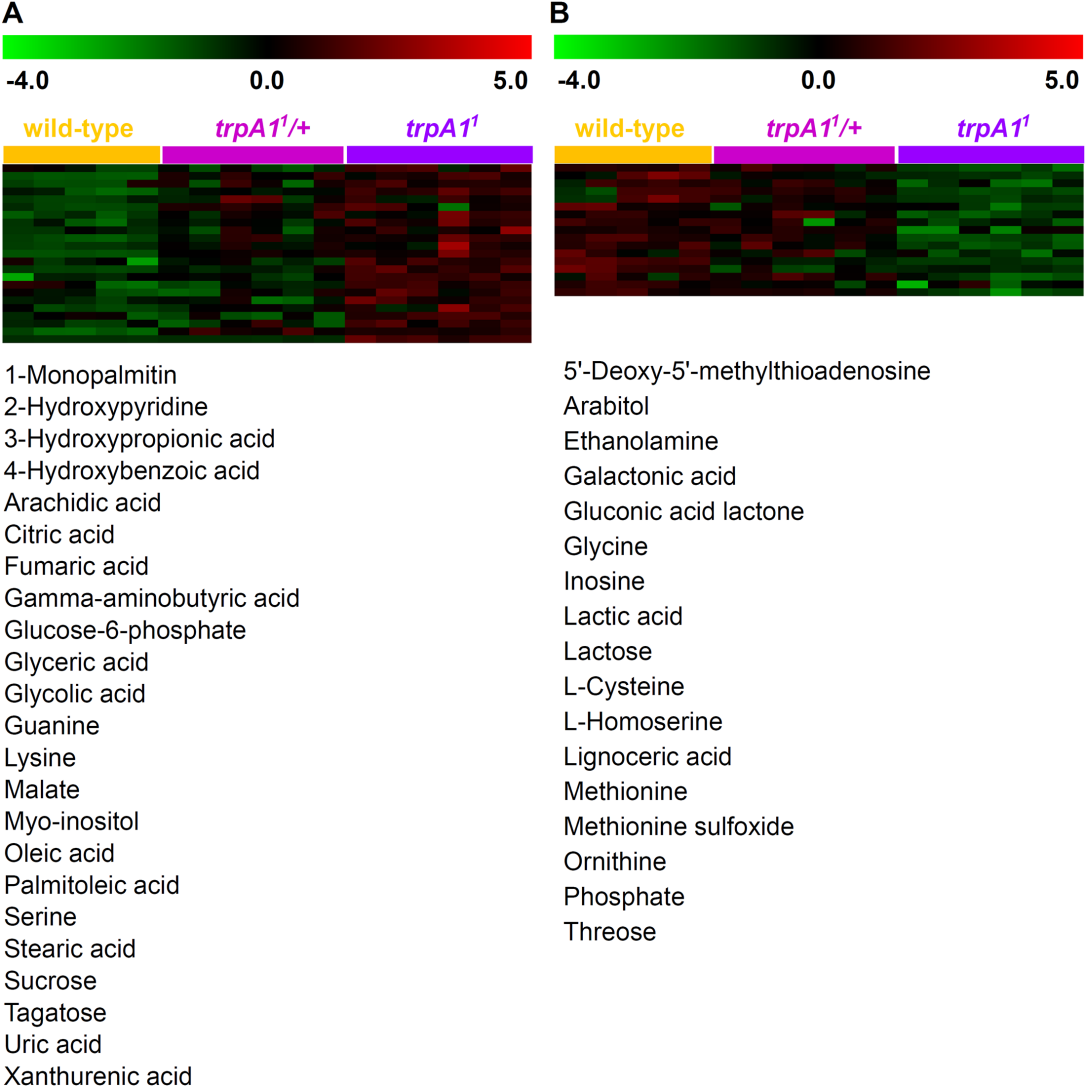
Gene-dosage effect of *trpA1* on the primary metabolism. Pavlidis template matching (PTM) analysis was performed to identify (A) the gradual increase and (B) decrease in metabolite abundances.

First, we examined the gene dosage-dependent alteration in metabolite abundances with the wild-type, heterozygotes (*trpA1*^*1*^/+), and homozygous mutants (*trpA1*^*1*^). A mild genetic perturbation using gene-dosage variation is a valuable tool for investigating intricate molecular networks [38]. The tracking of consequential changes in metabolite expression patterns following variation in gene dosage may narrow down the identification of factors directly linked to the function or interactive outcome of *trpA1*. For detecting gene dosage effects corresponding to the co-regulatory pattern in metabolite abundances, we performed Pavlidis template matching (PTM) analysis [39], which allows for capturing “gradual increase or decrease” in metabolites abundances. The cluster analysis identified two major clusters (Fig 4). The first cluster consisted of 17 metabolites showing a dosage-dependent down-regulation pattern. Note that among them, the intermediates of cysteine-methionine metabolites, including methionine, cysteine, 5-methylthioadenosine, homoserine, and methionine sulfoxide, are remarked in the reconstructed metabolic network. The second cluster presented the group of metabolites with a gradual up-regulation in accordance with the *trpA1* expression level (wild-type *<* heterozygotes *<* homozygotes). The cluster was composed of 1-monopalmitin, 2-hydroxypyridine, 4-hydroxybenzoic acid, GABA, malate, oleic acid, serine, tagatose, and xanthurenic acid. Unlike the down-regulated cluster, the gene dosage-dependent up-regulation did not clearly define metabolic and/or biochemical relevance among the metabolite clustered.

Second, we validated the consensus for metabolic changes by pair-wise analysis of metabolite changes in other genetic settings, including wild-type, *trpA1*^*1*^, *UAS*-*trpA1*;*trpA1*^*GAL4*^, and *trpA1*^*GAL4*^, which led to exclusive identification of the metabolic impact of *trpA1*. *trpA1*^*GAL4*^ is an additional allele in which the N-terminal 61 residues are deleted by the insertion of GAL4 in second ATG starting codon; it allows us to test for the rescue of the mutant phenotype with a *UAS*-*trpA1* transgene [9]. This allele is also a functional null, because the phenotypic defects of *trpA1*^*GAL4*^ share the same level of defects in *trpA1*^*1*^ in chemosensensation [9]. In addition, other group also shows the same aberrant defect in defecation regulation with *trpA1*^*GAL4*^ and *trpA1*^*1*^ by the expression of TRPA1 in a subset of enteroendocrine cells [40]. However, we do not exclude the possible undetectable expression of other transcript in *trpA1*^*GAL4*^.

So we inspected whether there were genotype-specific metabolic regulations between different alleles. Thus, we directly compared the metabolites that were significantly different between *trpA1*^*1*^ and *trpA1*^*GAL4*^. The result revealed only three metabolites including cyano-L-alanine, xylose, and tyrosine that showed statistical significance. The ratio (the number of significantly different metabolite-to-total variable number, 3/109, 2.8%) did not exceed 5.0% (based on the statistical criteria, *P*<0.05), which may fall into systematic error range instead of indicating genuine differences induced by different isoforms variance.

**Fig 5:**
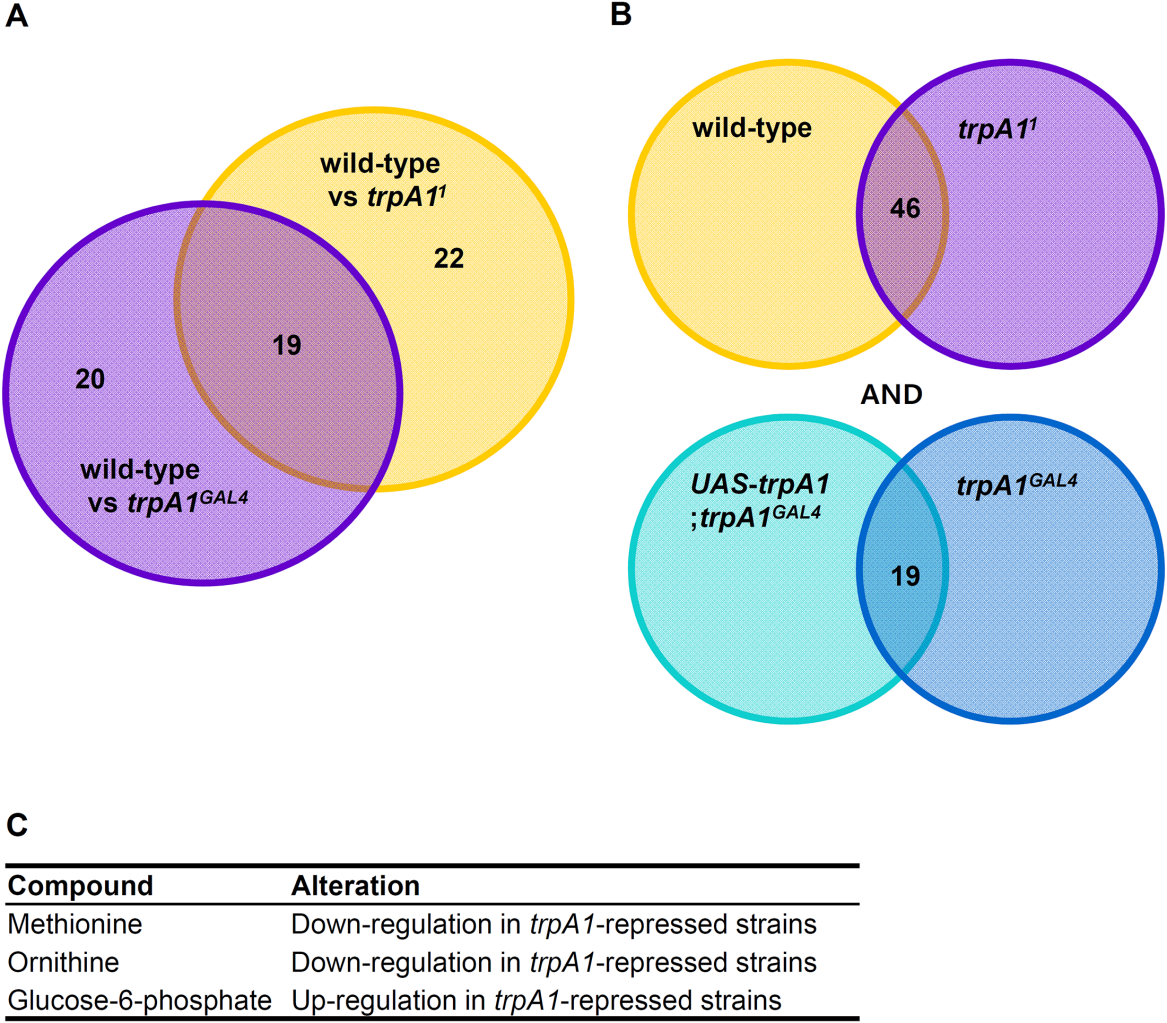
Metabolites showing consensus expression pattern across different genetic settings. (A) Venn diagram showing allele-specific metabolite changes in *trpA1*^*1*^ and *trpA1*^*GAL4*^ respectively, compared with wild-type (B) Venn diagram showing the chemical overlap between *trpA1*-expressed strains and *trpA1*-repressed strains along with two independent comparisons. (C) List of metabolites that are coordinately altered according to *trpA1* gene expression.

Alternatively we compared the metabolite lists, which was significantly different in each mutant type compared to the wild-type (e.g. *trpA1*^*1*^ vs wild-type, and *trpA1*^*GAL4*^ vs wild-type), and selected only the unshared metabolite lists between these two groups (Fig 5A). The metabolites, which showed a unique difference only in *trpA1*^*1*^, included 22 compounds, while *trpA1*^*GAL4*^-specific alteration was observed in 20 metabolites. The consequent pathway analysis revealed that *trpA1*^*1*^ was relatively unique to fatty acid and carbohydrate metabolisms while *trpA1*^*GAL4*^ was more relevant to metabolic dysregulation in amino acid metabolism. Although the comparison between different mutant isoforms suggested the probability of *trpA1* isoform-specific metabolic regulation, we concluded the potential difference did not affect the statistical result in the current study.

**Fig 6:**
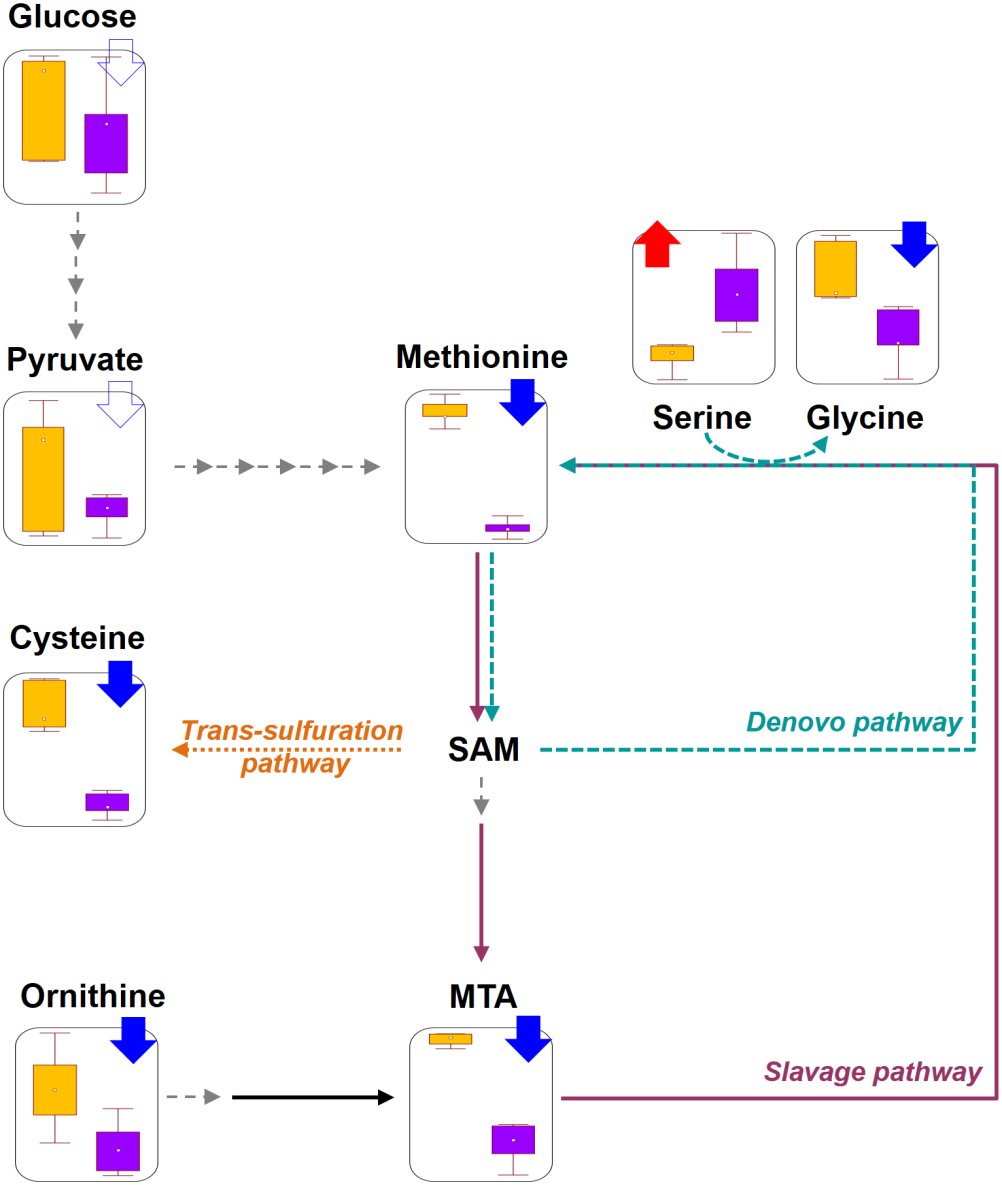
Proposed functional linkage of *trpA1* to central carbon metabolism and methionine salvage pathway in *Drosophila*. Arrows with filled colors indicate significant changes (*P*<0.05) in *trpA1* mutants compared to wild-type, and arrows without any colors presented the down-regulation pattern but without statistical significance. Data distributions were displayed by box-whisker plots, giving the mean value, the standard error as the box, and whiskers indicating 1.96 fold the standard.

We finally performed statistical comparisons between *UAS*-*trpA1*;*trpA1*^*GAL4*^ and *trpA1*^*GAL4*^, and investigated the common alteration that was compatible to the metabolite list from wild-type and *trpA1*^*1*^ comparisons (Fig 5B and 5C). The result showed that three metabolites were coordinately altered with equivalent expression patterns across the different gene settings. Glucose-6-phosphate showed significant increase in *trpA1*^*GAL4*^ mutants compared to its rescue strain, *UAS*-*trpA1*;*trpA1*^*GAL4*^, which corresponded to the up-regulation in *trpA1*^*1*^ as compared to the wild-type. Concurrently, two amino acids, methionine and ornithine presented significant down-regulation in *trpA1*^*GAL4*^ and was identical to the expression pattern from wild-type and *trpA1*^*1*^ comparison. The statistical readout through the pair-wise comparison across multiple gene setting suggested that the most probable metabolic targets of *trpA1* would be methionine and ornithine, which were consistently identified in the metabolic cluster map and the metabolite set with gene dosage-dependent changes. The molecular dynamics implies that synchronized metabolic modulation could be the outcome of a systematic re-organization of the methionine salvage pathway tightly coupled to the polyamine pathway [41] under a *trpA1*-deleted condition, as suggested in Fig 6.

One of the most well-known roles of TRPA1 is heat-sensing. Although we did not aim the discovery of thermal sensing functionality of TRPA1 in the current study, we alternatively explored the temperature-associated metabolic changes, which requires the experimental design with high temperature stress. First of all, the simultaneous changes in a range of fatty acids and lipid (1-monopalmitin) may alternatively imply the systematic alteration in membrane fluidity, which is commonly induced by abnormal temperature condition [42]. Further potential linkage was suggested from the significant up-regulation in myo-inositol and gamma-aminobutyric acid (GABA). The involvedness of myo-inositol has been proposed in primary protection against indirect temperature injury in Drosophila [43]. Likewise, the up-regulation of GABA could be linked to its potential role, the pain relieving process [44] and protection effect [45] against heat stress. In addition, trehalose, which is closely associated with heat protective cellular processes [46], was moderately up-regulated under a *trpA1*-deleted condition.
